# Optimization and validation of the international metabolic prognostic index for CD19 CAR-T in large B-cell lymphoma

**DOI:** 10.1038/s41408-025-01338-1

**Published:** 2025-08-26

**Authors:** Michael Winkelmann, Sandeep S. Raj, Michael D. Jain, Gloria Iacoboni, Fabian Müller, Leo Hansmann, Magdalena Corona, Alejandro Luna, Khushali Jhaveri, Gunjan L. Shah, Michael Scordo, Turab Mohammad, Erin A. Dean, Gabriel T. Sheikh, Wolfgang G. Kunz, Tobias Tix, Veit L. Bücklein, Akshay Bedmutha, Doris Leithner, Michael von Bergwelt-Baildon, Alexander P. Boardman, M. Lia Palomba, Jae H. Park, Gilles Salles, Miguel-Angel Perales, Heiko Schöder, Marion Subklewe, Pere Barba, Frederick L. Locke, Roni Shouval, Kai Rejeski

**Affiliations:** 1 Department of Radiology, LMU University Hospital, LMU Munich, Munich, Germany; 2 German Cancer Consortium (DKTK), Partner Site Munich, a partnership between the DKFZ Heidelberg and LMU University Hospital, Munich, Germany; 3 Adult Bone Marrow Transplantation Service, Department of Medicine, Memorial Sloan Kettering Cancer Center, New York, NY USA; 4 Department of Blood and Marrow Transplant and Cellular Immunotherapy, Moffitt Cancer Center, Tampa, USA; 5 Department of Hematology, University Hospital Vall d’Hebron, Vall d’Hebron Institute of Oncology (VHIO), Barcelona, Spain; 6 Department of Medicine, Universitat Autònoma de Barcelona, Bellaterra, Spain; 7 Bavarian Cancer Research Center (BZKF), partner sites Munich and Erlangen, Munich, Germany; 8 Department of Internal Medicine 5 - Hematology and Oncology, University Hospital of Erlangen, Friedrich-Alexander-Universität Erlangen-Nürnberg, Erlangen, Germany; 9 Department of Internal Medicine III, University Hospital Regensburg, Regensburg, Germany; 10 Division of Hematology/Oncology, IU Simon Comprehensive Cancer Center, Indiana University School of Medicine, Bloomington, USA; 11 Department of Medicine, Weill Cornell Medical College, New York, NY USA; 12 Department of Medicine, Division of Hematology/Oncology, University of South Florida, Tampa, FL USA; 13 Division of Hematology and Oncology, Department of Medicine, University of Florida, Gainesville, FL USA; 14 Division of Nuclear Medicine, LMU University Hospital, LMU Munich, Munich, Germany; 15 Department of Medicine III – Hematology/Oncology, LMU University Hospital, LMU Munich, Munich, Germany; 16 Department of Radiology, Memorial Sloan Kettering Cancer Center, NY, USA; 17 Lymphoma Service, Department of Medicine, Memorial Sloan Kettering Cancer Center, NY, USA; 18 Leukemia Service, Department of Medicine, Memorial Sloan Kettering Cancer Center, NY, USA

**Keywords:** Risk factors, B-cell lymphoma

## Abstract

While CD19-directed CAR T-cell therapy represents a transformative immunotherapy for relapsed/refractory large B-cell lymphoma (r/r LBCL), more than 50% of patients ultimately progress or relapse. Recently, the International Metabolic Prognostic Index (IMPI) – incorporating age, stage, and metabolic tumor volume (MTV) – was shown to improve prognostication for LBCL frontline treatment. Here, we examine its utility to predict toxicity and survival in CAR-T recipients. This multicenter observational study spanning six international sites included 504 patients with available ^18^FDG-PET/CT imaging at last response assessment prior to lymphodepletion. Optimal CAR-adapted MTV thresholds were identified in a development cohort (*n* = 256) and incorporated into a CAR-T-specific IMPI (“CAR-IMPI”). The prognostic performance of CAR-IMPI was validated in an independent cohort (*n* = 248). CAR-IMPI risk categories, defined by the median (1.35) and terciles (1.07, 1.58), demonstrated significant discrimination for progression-free survival (PFS; *p* < 0.0001) and overall survival (OS; *p* < 0.0001) in both cohorts. Multivariate Cox regression confirmed CAR-IMPI as an independent predictor of survival, accounting for pre-lymphodepletion LDH and CRP, performance status, treatment center, and CAR-T product. Patients in the CAR-IMPI high-risk category experienced increased severity of CRS and ICANS, and higher rates of intensive care unit (ICU) admissions. In an exploratory analysis, combining CAR-IMPI with established indices of high-risk systemic inflammation (CAR-HEMATOTOX, InflaMix) further enhanced survival stratification. The CAR-IMPI may provide a potent and validated PET-based tool for risk stratification of clinical outcomes in patients with r/r LBCL receiving CD19 CAR-T therapy. Our data highlight the utility of combining clinical and radiological modalities, with implications for patient selection and the anticipated level-of-care for toxicity management.

## Introduction

Chimeric antigen receptor T-cell (CAR-T) therapy targeting the CD19 antigen represents an established treatment for patients with relapsed/refractory large B-cell lymphoma (r/r LBCL), resulting in significant improvement of survival outcomes [1–7]. Still, more than 50% of CAR-T recipients ultimately do not achieve a durable response. In addition, CAR T cells induce unique side effects, including cytokine release syndrome (CRS) and immune effector cell-associated neurotoxicity syndrome (ICANS), which can result in ICU admissions and contribute to morbidity and mortality [8–13].

The efficacy of CAR-T therapy is impacted by several factors [14]. Host factors include performance status, hematopoietic reserve, and gut microbiome composition [15–19]. Product attributes relate to the quality and fitness of T cells obtained for manufacturing, the subsequent CAR T-cell expansion kinetics, and CAR T-cell persistence [20]. Genomic alterations of the underlying lymphoma (e.g., TP53 mutations, copy number changes, chromosomal instability) and the tumor immune environment are additional response determinants, which can be associated with the metabolic tumor volume (MTV) as determined by ^18^FDG-PET/CT imaging [21–29]. Classic prognostic tools like the revised International Prognostic Index (IPI) integrate readily available clinical parameters (age, stage, ECOG, LDH, extranodal involvement), but have modest utility in the context of CAR-T therapy [7, 22, 30]. In contrast, inflammation-based scores like the CAR-HEMATOTOX [16, 31, 32] or InflaMix [33] may better reflect the immunohostile micromilieu that drives poor treatment outcomes in lymphoma patients receiving CD19 CAR-T [26, 34]. Importantly, both tumor burden and the systemic inflammatory state have also been linked to the development of key CAR-T toxicities [13, 35, 36].

Recently, the International Metabolic Prognostic Index (IMPI) was introduced as a novel prognostic index for LBCL patients undergoing first-line Rituximab-based treatment and was reported to have superior discriminating power than the conventional IPI [37]. The IMPI provides continuous and individualized survival estimates and consists of three factors: MTV, age and Ann Arbor stage. Another study examined the IMPI for first-line therapy in a real-world setting, but showed non-superior diagnostic performance compared with the conventional IPI and NCCN-IPI [38]. In the CAR-T context, we previously published a single-center pilot study of 39 patients showing a modest association with progression-free (PFS), but not overall survival (OS) [39]. This raised the question if MTV thresholds derived in the first-line setting are applicable to r/r LBCL patients, or if further modifications of the IMPI are necessary for CAR-T recipients. Furthermore, the external validity of the IMPI scoring system remains unclear, particularly considering regional differences in CAR-T delivery across geographic regions (e.g., differences in vein-to-vein intervals, tumor burden) [40, 41].

To address these gaps, we aimed to optimize and validate IMPI in a large multicenter international cohort of patients treated with CD19 CAR-T. Following development and validation of a CAR-adapted IMPI, we assessed its association with key toxicity and survival outcomes. As a secondary objective, we evaluated whether inflammation-based scores (e.g., CAR-HEMATOX, InflaMix) supplement survival prognostication with CAR-IMPI.

## Methods

### Study design and population

This multicenter study incorporated r/r LBCL patients treated with standard-of-care CD19 CAR-T products between 2017 until June 2023.

The following inclusion criteria were applied:

1. Patients with r/r LBCL(2nd line and above),
2. Available pre-therapeutic ^18^F-FDG PET/CT imaging studies,
3. Available information regarding age and Ann Arbor stage.

The following exclusion criteria were applied:

1. Missing or incomplete baseline imaging,
2. Outlier value for MTV (highest 2.5% of measurements).

Patients treated with CD19 CAR-T across five centers (Moffitt, Erlangen, Regensburg, LMU Munich, Vall d’Hebron Barcelona) were included in the development cohort, while those treated at Memorial Sloan Kettering were used for validation. All medical records and imaging studies were reviewed with Institutional Review Board approval and informed patient consent was obtained. Patients received lymphodepletion according to the manufacturer’s instructions [2, 4, 42].

### Development of the CAR-adapted IMPI

We extracted the IMPI variables outlined by Mikhaeel et al (Fig. S1) [37]. MTV and Ann Arbor stage were measured from ^18^F-FDG PET/CT imaging at last response assessment prior to CAR-T infusion using LIFEx or MIM software. MTV segmentation was performed using an absolute SUV threshold of 4.0 for patients from LMU Munich and MSKCC, and with a fixed threshold of 41% of the SUVmax for all other patients, as previously described [43]. Age was calculated on the day of infusion. Similar to the development of the original IMPI, we first fit a restricted cubic spline function to describe the relationship between the hazard of death or progression and MTV in the model development cohort. To identify the optimal MTV cutoff value, we used maximally selected rank statistics for PFS (*maxstat* R package). Using the development cohort, we then fit a Cox proportional hazards regression model using a linear spline of MTV (cutpoint at 44.3 mL), age, and stage as predictors. Model coefficients for each variable were used to determine linear predictors, which defined CAR-adapted IMPI scores (detailed formula found in Supplemental Methods). CAR-adapted IMPI scores were stratified by median and into low, intermediate, and high-risk groups by terciles in the development cohort. The same cutoff values defined risk groups in the validation cohort.

### Calculation of inflammation-based prognostic scores

Baseline laboratory markers were extracted prior to lymphodepletion with a leniency period of up to 5 days. The CAR-HEMATOTOX score was calculated using hemoglobin, platelet count, absolute neutrophil count (ANC), CRP, and ferritin [31]. Because of the improved prognostic capacity of a higher score threshold in the original publication [31], a CAR-HEMATOTOX cutoff of 3 was used. To assign the “inflammatory” versus “non-inflammatory” InflaMix clusters, we applied the previously outlined unsupervised machine learning approach [33]. Cluster assignment was based on a maximum of 14 available pre-infusion laboratory and cytokine measurements.

### Statistical Analysis

PFS was defined from CAR-T infusion until progression of lymphoma was detected on (PET/)CT as defined by Lugano criteria, or death [44]. OS was defined from the day of CAR-T infusion until death from any cause. Kaplan-Meier estimates were used to generate survival curves. Log-rank test was performed to examine the significance of the results. Statistical analyses and visualization were performed using R Project (v4.4.2). Uni- and multivariable Cox regression analyses studied the association of CAR-IMPI and key patient-, disease-, and treatment-related confounders with PFS and OS. Mann-Whitney U, Kruskal-Wallis, Fisher’s exact test and Chi-square were used to test the statistical significance of differences in clinical parameters between CAR-IMPI risk groups. *P* values < 0.05 were considered to indicate statistical significance.

## Results

### Patient characteristics

In total, 504 patients met study inclusion criteria, including 256 patients in the development cohort and 248 patients in the validation cohort. Median age was 65 years, 37% of patients were female, and 24% of patients had an ECOG performance status of 2 or greater (Table 1). The majority of patients received axicabtagene ciloleucel (axi-cel, 57%), followed by tisagenlecleucel (tisa-cel, 30%) and lisocabtagene maraleucel (liso-cel, 14%). Patients had received a median of 2 (IQR 1–3) lines of prior systemic therapy, including 22% of patients with a prior autologous hematopoietic cell transplantation (HCT). On the last PET imaging, the median MTV was 49 mL (IQR 5–223 mL), and most patients presented with advanced disease, reflected by an Ann-Arbor stage of 3 or higher (74%). Primary refractory disease and transformed lymphoma were noted in 40% and 32% of cases, respectively.

**Table 1:** Patient Characteristics.

	All patients (*n* = 504)^a^	Development (*n* = 256)	Validation (*n* = 248)
**Patient demographics**			
Median age (range)	65 (56, 71)	64 (57, 70)	66 (56, 73)
Sex			
Female	188 (37%)	99 (39%)	89 (36%)
Male	316 (63%)	157 (61%)	159 (64%)
ECOG Score			
>1	70 (14%)	36 (14%)	34 (14%)
0-1	434 (86%)	220 (86%)	214 (86%)
**Treatment-related features**			
CAR-T Product			
Axicabtagene ciloleucel	285 (57%)	155 (61%)	130 (52%)
Lisocabtagene maraleucel	70 (14%)	19 (7.4%)	51 (21%)
Tisagenlecleucel	149 (30%)	82 (32%)	67 (27%)
Number of prior treatment lines (excluding Bridging)	2 (1, 3)	2 (2, 3)	2 (1, 3)
Received bridging therapy	386/502 (77%)	190/254 (75%)	196 (79%)
**Disease features**			
Primary refractory disease	202/503 (40%)	101 (39%)	101/247 (41%)
Post-bridging bulky disease ( > 10 cm)	46/436 (11%)	29/253 (11%)	17/183 (9.3%)
Baseline MTV	49 ml (5, 223)	68 ml (12, 306)	24 ml (1, 156)
Prior Autologous HCT	111 (22%)	59 (23%)	52 (21%)
Ann Arbor Stage			
0-2	133 (26%)	58 (23%)	75 (30%)
3-4	371 (74%)	198 (77%)	173 (70%)
Transformed Disease	159/503 (32%)	68/255 (27%)	91 (37%)
Double/Triple Hit	109/364 (30%)	74/137 (54%)	35/227 (15%)
**Laboratory Findings (Pre-LD)**			
Baseline LDH (U/L)	253 (193, 400)	275 (211, 455)	230 (185, 362)
Baseline Hemoglobin (g/dL)	10.5 (9.3, 11.8)	10.6 (9.3, 11.8)	10.5 (9.1, 11.9)
Baseline Platelets (10^9^/L)	168 (113, 219)	165 (108, 219)	169 (114, 219)
Baseline ANC (10^9^/L)	3.10 (1.91, 4.60)	2.80 (1.70, 4.40)	3.40 (2.15, 4.80)
Baseline Ferritin (ng/mL)	349 (107, 812)	447 (211, 1026)	226 (74, 549)
Baseline CRP (mg/dL)	1.0 (0.4, 3.4)	1.3 (0.4, 4.0)	0.8 (0.4, 3.0)
Hematotox Score			
High ( ≥ 3)	132 (27%)	74 (30%)	58 (23%)
Low (0–2)	362 (73%)	172 (70%)	190 (77%)
Unknown^b^	10	10	0

^a^Median (Q1, Q3); n (%). The denominator has been included in case of missing values.

^b^CRP and Ferritin values missing in five patients in the development cohort, respectively. CAR-HEMATOTOX scores were evaluable in 494 patients and calculated according to *Rejeski et al, Blood 2021*; a higher score threshold of 3 was utilized, based on its improved prognostic capacity in the original publication.

*ECOG* Eastern Cooperative Oncology Group, *MTV* metabolic tumor volume, *HCT* hematopoietic cell transplantation, *LD* lymphodepletion (typically day -5 before CAR T-cell infusion), *LDH* Lactate Dehydrogenase, *ANC* absolute neutrophil count, *CRP* C-reactive protein.

With a median follow-up of 32.1 months (development [D]: 27.6, validation [V]: 45.4 mo, Table S1), median PFS was 7.6 months (D: 7.9, V: 7.5 mo) and median OS was 34.0 months (D: 33.4, V: 34.0 mo). When comparing patient features, the distribution of age, gender, ECOG, number of previous therapy lines, and rate of bridging therapies was comparable across both cohorts. The validation cohort had a higher proportion of patients treated with liso-cel (21 vs 7.4%). In contrast, patients in the development cohort at baseline showed a higher median MTV (68 vs 24 mL), higher median serum LDH (275 vs 230 U/L) and higher serum inflammatory markers (Table 1).

### Optimizing the MTV threshold for CAR T-cell therapy

We hypothesized that, although the individual components of the IMPI score are informative for outcomes following CAR-T therapy, the MTV cutoff used in regression modeling could be further optimized for pre-CAR-T risk stratification. To investigate this, we first assessed in the training cohort whether the association between MTV and the hazard of death or disease progression after CAR-T infusion resembled the relationship observed in first-line lymphoma therapy. A monotonic relationship was observed, with higher MTV values corresponding to an increased hazard of death or progression. The graphical inflection point was identified at an MTV of ~50 mL (Fig. S2A), which was markedly lower than observed for the original IMPI in first-line LBCL (307.9 mL) [37].

**Fig. 1:**
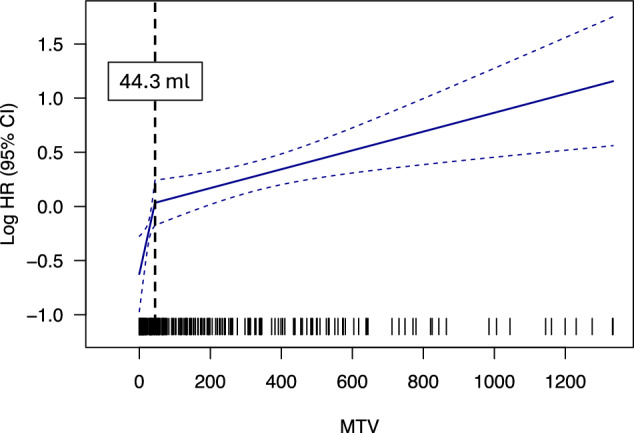
Linear Spline with one knot. Depicted are the results of a linear spline model with one knot for the CAR-T cell-specific metabolic tumor volume (MTV) threshold determined in the development cohort. MTV is plotted on the x-axis and the log-transformed hazard ratio (HR) on the y-axis.

To refine the MTV cutoff that best captures the relationship between MTV and the risk of death or progression, maximally selected rank statistics were applied, identifying an optimal threshold of 44.3 mL (Fig. S2B). The hazard ratio (HR) for PFS increased more sharply for patients with MTV values below this threshold (log HR increased by 1.49 per 100-ml increase in MTV) compared to those with MTV above this threshold (log HR increased by 0.87 per 100 ml increase in MTV) (Fig. 1). These findings underscore the need to interpret and model MTV differently for patients undergoing CAR-T therapy compared to those receiving frontline treatment. Therefore, the optimized MTV cutoff was subsequently incorporated into a CAR-adapted IMPI formula, which was derived using the study’s development cohort.

### CAR-adapted IMPI (CAR-IMPI) and survival

**Fig. 2:**
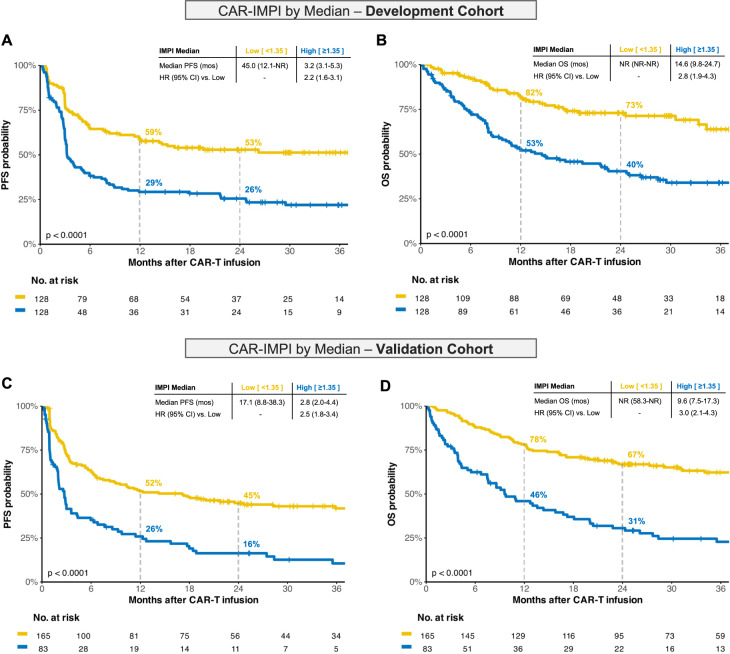
Survival Analysis stratified by Median CAR-IMPI and Transfer to the Validation Cohort. The upper panel shows the Kaplan-Meier curves for progression-free survival (PFS; **A** and overall survival (OS; **B** for development cohort stratified by median CAR-IMPI. The lower part of the figure displays PFS (**C**) and OS (**D**) survival curves for validation cohort using the same CAR-IMPI cut-off established in the development cohort. The low risk group is marked in yellow and the high risk group in blue. The hazard ratio (HR) with 95% confidence interval (CI) from the univariate Cox regression using the low-risk group as reference is provided.

We first evaluated the prognostic value of the CAR-adapted IMPI (CAR-IMPI) for PFS and OS in the development cohort. Stratifying the development cohort by median CAR-IMPI (1.35) yielded significant differences in estimated PFS (Fig. 2A) and OS (Fig. 2B). Furthermore, subdivision into three equal risk groups based on CAR-IMPI terciles—reflecting expected response rates in the relapsed/refractory setting [41, 45]—effectively stratified for PFS and OS (Figure S3A,B). In the low-risk group (CAR-IMPI < 1.07), median PFS was 45.0 (95% CI 19.6–not reached) months. The intermediate-risk group (CAR-IMPI 1.07–1.53) had a median PFS of 8.9 (95%CI 4.0–24.8) months. High-risk patients (CAR-IMPI > 1.53) had poor outcomes, with a median PFS of only 3.1 (95%CI 2.8–4.0) months (Fig. S3A).

### Independent validation of the prognostic capacity of CAR-IMPI

To verify the above results, the CAR-IMPI cut-off values for median and the three risk groups determined in the development cohort were tested in the independent validation cohort. Reflecting their decreased MTV values at last response assessment, we noted lower CAR-IMPI scores in the validation cohort (1.07 vs. 1.36, *p* < 0.001; Figure S4) and more patients were thus assigned to the ‘low-risk’ group (48 vs. 33%). In the case of a median split, the validation cohort showed significant discrimination for both PFS (Fig. 2C) and OS (Fig. 2D). Patients with above-median CAR-IMPI had significantly shorter PFS (HR 2.5, 95% CI 1.8–3.4) and OS (HR 3.0, 95% CI 2.1–4.3) compared to the patients with a below-median CAR-IMPI. Furthermore, the three CAR-IMPI-based risk groups displayed a marked stratification of the survival curves (Fig. S3C, D). In the validation cohort, median PFS was 23.5 (95%CI 10.6–not reached) months for ‘low-risk’, 8.8 (95%CI 3.4–19.3) months for ‘intermediate-risk’ and only 2.0 (95%CI 1.0–2.8) months for ‘high-risk’ patients.

### Multivariate Cox regression analysis

Among considered baseline features, high CAR-IMPI scores were associated with increased ECOG and higher serum LDH and CRP values (Table S2). These differences were particularly evident for the patients in the upper-most CAR-IMPI tercile (Table S3). To account for these clinically relevant covariates and evaluate the independent prognostic capacity of CAR-IMPI, we performed multivariate Cox regression analyses. In addition to CAR-IMPI, pre-lymphodepletion LDH and CRP, ECOG performance status and CAR-T product were explored, as was the treatment center variable for the development cohort. Notably, CAR-IMPI was independently associated with PFS in the development (adjusted *p* = 0.002) and validation cohort (adjusted *p* = 1.9 × 10^-8^) (Table 2). Similarly, we found an independent association between CAR-IMPI and OS in the development (adjusted *p* = 0.006) and validation cohort (adjusted *p* = 1.3 × 10^-8^) (Table S4).

**Table 2:** Multivariate Cox Regression for Progression-Free Survival.

Characteristic	Development			Validation		
	N	HR (95% CI)	p	N	HR (95% CI)	p
**CAR-IMPI** (continuous)	256 (100%)	1.95 (1.28–2.99)	**0.002**	248 (100%)	1.71 (1.42–2.06)	**1.9** **×** **10**^**-8**^
**CAR-T Product**						
Axi-cel	155 (61%)	Ref.		130 (52%)	Ref.	
Liso-cel	19 (7.4%)	1.74 (0.90–3.35)	0.098	51 (21%)	1.71 (0.46–1.13)	0.157
Tisa-cel	82 (32%)	1.65 (1.09–2.49)	**0.018**	67 (27%)	2.04 (1.43–2.91)	**7.5** **×** **10**^**-5**^
**ECOG**						
0–1	220 (86%)	Ref.		163 (66%)	Ref.	
2–4	36 (14%)	1.46 (1.12–1.91)	**0.005**	85 (34%)	1.29 (0.92–1.80)	0.141
**LDH** ( > ULN)	256 (100%)	1.23 (0.81–1.86)	0.334	248 (100%)	1.58 (1.11–2.25)	**0.011**
**CRP** (continuous)	251 (98%)	1.01 (0.99–1.02)	0.365	248 (100%)	1.03 (1.00–1.07)	0.052
Center*	*	*	*	-	-	-

The output of the multivariable Cox Regression model for progression-free survival is provided for the development and validation cohort – performed separately. P-values reaching statistical significance (*p* < 0.05) are highlighted in bold. The number of patients (N) in each strata and respective reference (Ref.) variable are depicted. All laboratory values were determined before lymphodepletion with a leniency period of 5 days.

^*^ The center variable was introduced into the multivariable model as a stratification variable (not applicable to the monocentric validation cohort).

*HR* hazard ratio, *CI* confidence interval, *CAR* chimeric antigen receptor, *IMPI* international metabolic prognostic index, *Axi-cel* axicabtagene ciloleucel, *Liso-cel* lisocabtagen maraleucel, *Tisa-cel* tisagenlecleucel, *ECOG* Eastern Cooperative Oncology Group, *LDH* Lactate Dehydrogenase, *ULN* upper limit of normal, *CRP* C-reactive protein.

### CAR-IMPI and toxicity outcomes

**Fig. 3:**
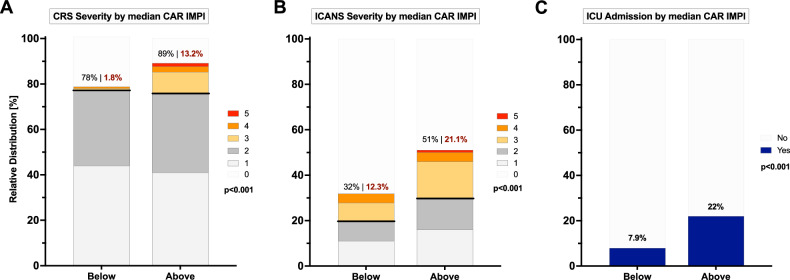
Frequency and Severity of CRS and ICANS by CAR-IMPI Risk Group. Depicted are with severity of cytokine release syndrome (CRS; **A** and Immune effector cell-associated neurotoxicity syndrome (ICANS; **B** and the probability of ICU admission (**C**) by median CAR-IMPI. The stacked bar plots show the total number of patients. The p-values indicate the results of the Chi-Square and Fisher’s exact test.

Next, we examined the relationship between CAR-IMPI and key toxicity outcomes. We did not find significant differences in CRS or ICANS severity between the development and validation cohorts (Table S1), which were subsequently combined for the safety analysis. We observed a significant increase in high-grade CRS (ASTCT grade ≥3°) in the above-median CAR-IMPI group (13.2 vs. 1.8%, *p* < 0.001, Fig. 3A). Furthermore, patients with higher CAR-IMPI values more commonly developed high-grade ICANS (21.1 vs. 12.3%, Fig. 3B) and ICU admissions were more frequent (22 vs. 7.9%, Fig. 3C). Of note, the patients with the highest CAR-IMPI values (upper tercile) carried a particularly high-risk for severe toxicity and showed increased utilization of supportive therapies like tocilizumab and corticosteroids (Table S5).

### Combined radio-inflammatory indices refine prognostication in patients receiving CD19 CAR-T therapy

**Fig. 4:**
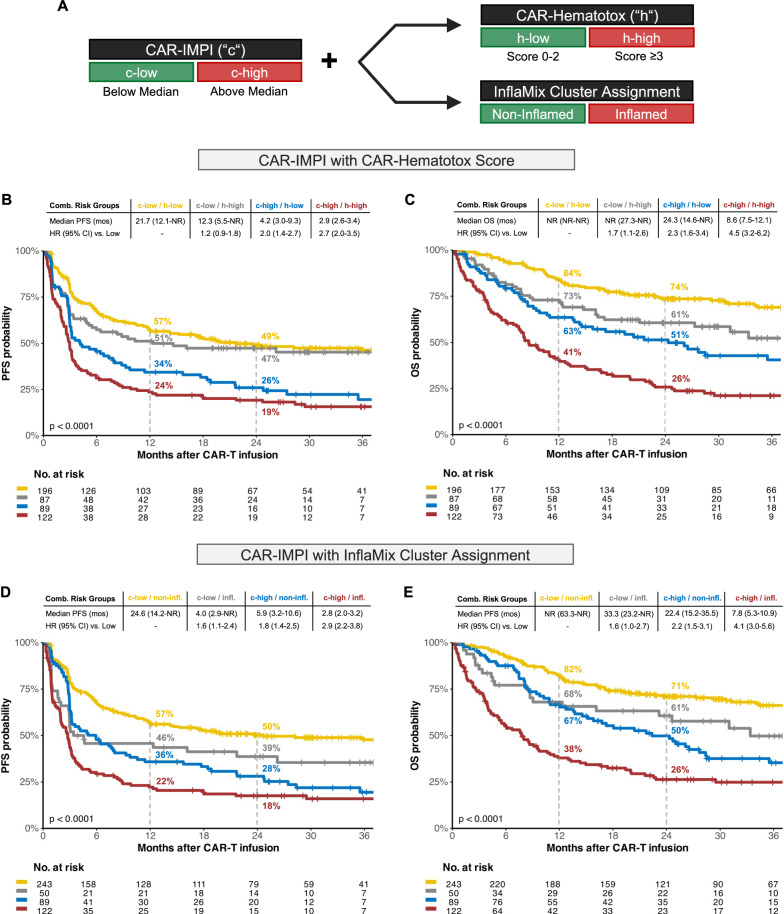
Modification of CAR-IMPI with CAR-HEMATOTOX and InflaMix Cluster Assignment. Illustrated are Kaplan-Meier survival curves for CAR-IMPI risk groups modified by CAR-HEMATOTOX and InflaMix. **A** Schema showing the combination of CAR-IMPI (stratified by median) and inflammation-based risk scores. CAR-HEMATOTOX high is defined for patients with values ≥ 3 and low with values ≤ 2. **B**, **C** Estimated progression-free (PFS, **B**) and overall survival (OS, **C**) by CAR-IMPI with CAR-HEMATOTOX. **D**, **E** Estimated progression-free (PFS, **D**) and overall survival (OS, **E**) by CAR-IMPI with Inflamix cluster assignment. Patients with low values in both parameters are marked in yellow, with high values in both parameters in red, the CAR-IMPI high and CAR-HEMATOTOX/InflaMix low-risk group in blue and CAR-IMPI low and CAR-HEMATOTOX/InflaMix high-risk group in gray. P-values by logrank test are provided in the graph inset. Median PFS or OS in months with the 95% confidence intervals are depicted above the Kaplan-Meier graph together with the results of the univariate Cox regression using the lowest-risk group as reference.

To evaluate whether inflammation-based scores can supplement prognostication of survival in CAR-T recipients, we integrated CAR-IMPI with the pre-therapeutic CAR-HEMATOTOX and InflaMix scores [16, 31]. For this exploratory analysis, data from both cohorts were pooled, and patients were categorized based on their median CAR-IMPI score (c-high: ≥1.35; c-low: <1.35, Fig. 4A). These groups were further stratified by CAR-HEMATOTOX scores (h-high: ≥3; h-low: 0–2) and InflaMix cluster assignment (inflamed vs. non-inflamed). The combined subgroups were then analyzed for PFS (Fig. 4B, D) and OS (Fig. 4C, E).

As expected, the lowest-risk patients (“c-low/h-low” or “c-low/non-inflamed”) showed the longest survival (1-year PFS for both 57%, 1-year OS 82 and 84%, respectively). Intermediary outcomes were noted for the subgroups in which only one of the parameters was considered high-risk. Within the intermediate groups, however, patients with a low CAR-IMPI but high CAR-HEMATOTOX score (“c-low/h-high”) exhibited improved survival compared to their “c-high/h-low” counterparts (1-year PFS: 51 vs. 34%, 1-year OS: 73 vs. 63%, Fig. 4B, C). For the combination of CAR-IMPI and InflaMix, similar survival curve trajectories within the intermediary groups manifested with extended follow-up (Fig. 4D, E). Notably, the group deemed high-risk by both radio-inflammatory indices (“c-high/h-high” or “c-high/inflamed”) – comprising ~25% of the total study cohort – showed markedly inferior survival outcomes (1-year PFS 22 and 24%, 1-year OS 38 and 41%).

## Discussion

In this multicenter international study, we established the utility of the individualized IMPI prognostication tool for treatment failure in patients receiving CD19 CAR-T therapy and refined the MTV threshold for its optimal use in r/r LBCL patients. CAR-IMPI was also associated with the severity of CRS and ICANS and the need for ICU admissions. Finally, we found that integration of inflammation-based scores with CAR-IMPI was able to refine prognostication of survival outcomes.

IMPI was originally developed to assess outcomes of LBCL patients undergoing first-line immunochemotherapy. It was demonstrated that the predictive power of the IMPI for PFS and OS was superior to that of the conventional IPI. Due to its recent publication, literature validating the IMPI remains limited. One study attempting to validate the IMPI in a collective of LBCL patients undergoing first-line treatment showed limited reproducibility [38]. A monocentric study investigating IMPI in the context of CAR-T therapy in a small cohort showed differences in PFS, but no significant differences in OS [39]. To our knowledge, this represents the first study to comprehensively investigate and externally validate a tumor metabolic prognostic index adapted to CAR-T therapy in a large multicenter cohort.

The MTV threshold for the risk calculation of the original IMPI is 307.9 ml, which corresponds to the median MTV of the cohort in which it was developed. In the CAR-T context, the MTV levels and cut-off values that have been described to be associated with adverse treatment outcomes in previous studies were lower. For example, different groups have reported varying MTV thresholds ranging from 24, 60, 80, and 147 ml, respectively [23, 46–48]. In our study, the median MTV was 49 ml and the CAR-adapted cut-off for high-risk patients was 44.3 ml, which both are lower than the reported values for first-line therapy and in a similar range with the values reported to be prognostic in the setting of CD19 CAR-T therapy [23, 46–48]. The development cohort had a higher median MTV compared to the validation cohort (68 vs. 24 ml) and displayed higher serum LDH values—likely reflecting cohort-level differences in underlying tumor burden. While serum LDH and MTV can both serve as surrogates of tumor burden and share similar prognostic information, combining both parameters can yield additional value in survival prediction [48]. Future studies may yet investigate the prognostic role of other PET-based metrics such as “SDmax/bulk” in combination with radiomic features like MTV, SUVpeak and Dmaxbulk and patient-related parameters like performance status and age [49, 50].

While IMPI provides individualized estimates of patient outcomes, the original publication divided patients into three groups for survival analysis: 10% at highest risk, 30% intermediate risk, and 60% lowest risk, corresponding with expected clinical outcome to first-line therapy. These groups also risk-stratified for PFS in a small cohort of patients with CAR T-cell treatment, but did not show significant differences in OS [39]. In our study, we found that dividing patients into three equally sized risk groups based on CAR-IMPI terciles discriminated for survival outcomes in the CAR-T context. This distribution is also more aligned with the clinical response rate of LBCL patients undergoing CAR-T therapy and could represent a useful modification in clinical practice. For example, real-world data from patients in Europe and the US indicate an objective response in approximately two-thirds of patients (of which ~1/3 remain durable at one year) and primary refractory cases in the remaining third (e.g., CAR non-responders) [6, 41, 45].

In the multivariate analysis, we confirmed that increasing CAR-IMPI scores were associated with poor survival even when accounting for other key prognostic factors. By integrating CAR-HEMATOTOX or InflaMix, we were able to further refine which patients carry the highest risk with CD19 CAR-T therapy. Prior studies have reported that the inflammatory markers incorporated in these scores more fundamentally reflect an immunohostile micromilieu and hint at the type of systemic immune dysregulation that blunts CAR T-cell expansion and portends poor treatment responses [16, 31, 34, 51]. This includes the upregulation of soluble T-cell checkpoint ligands and markers of macrophage activation, as well as increased suppressive myeloid cells and tumor interferon signaling [26, 27, 52, 53]. Overall, the excellent discrimination for survival achieved by combining CAR-IMPI and inflammation-based scores suggests that inflammatory markers can add supplemental value to tumor burden. It should also be noted that the CAR-HEMATOTOX and InflaMix models include markers of hematopoietic reserve, and that some of the differences in survival may be driven by severe cytopenias (ICAHT [54–56]) that can predispose for infections [16] and infection-driven NRM [9]. Ultimately, incorporating multiple, orthogonal data sources like labs and imaging studies represents a path forward to further improve prognostication of survival in LBCL patients receiving modern immunotherapies like CAR-T therapies or bispecific antibodies.

Our study has several limitations that need to be carefully considered when interpreting the results. First, it is limited by its retrospective design and only includes patients who actually received their manufactured CAR T cells. Second, MTV was calculated locally using different machines and software. Additionally, two distinct segmentation techniques were applied across centers. While these methods demonstrate a high correlation and similarly strong prognostic value for PFS in LBCL in the literature, segmentation using the 41% SUVmax threshold tends to yield slightly lower MTV values [43]. While this reflects real-world practice, this may have introduced heterogeneity to MTV measurements. Third, resulting from the operational and logistical nature of CAR-T, the clinical use of bridging therapy may affect the calculation of MTV as metabolic activity is likely altered by (effective) systemic bridging regimens [57]. Finally, it should be noted that age represents a component of the IMPI and is considered a negative prognostic factor. However, older CAR-T recipients (>65 years) deemed CAR-T eligible, have displayed encouraging response rates in some observational studies [6, 7, 58–60]. In addition, the negative prognostic impact of advanced age has not been shown for CAR-T therapy thus far in clinical trials like ALYCANTE or PILOT [61, 62]. To maintain the principal structure of the IMPI, both adaption of MTV and an adjusted *β*-coefficient for age in the formula to create a more CAR-specific IMPI (as was pursued in this study) represent reasonable approaches for elderly patients receiving CD19 CAR-T therapy.

Nonetheless, we see several salient clinical implications of CAR-IMPI. Advantages include its general capacity to provide more individualized and dynamic survival estimates. The additive properties of CAR-IMPI and inflammatory scores in refining prognostication of CAR-T outcomes are intriguing and point towards a model wherein tumor bulk and a (pathologic) state of systemic inflammation hamper effective clearance of the lymphoma by effector cells. Clinical implications relate to patient selection, particularly for identifying candidates for novel prophylactic combinatorial strategies to cytoreduce tumors and resolve systemic inflammation and/or consolidative therapeutic approaches. While patients with high CAR-IMPI scores may be triaged for inpatient admission due to concern for significant toxicity, low scores may help to guide the decision for outpatient CAR-T administration [63].

In conclusion, CAR-IMPI could represent a potent and validated PET-based tool for early risk stratification in r/r LBCL patients treated with CD19 CAR-T therapy. In addition to significant differences in PFS and OS between the CAR-IMPI-based risk groups, we observed higher CRS and ICANS severity and increased ICU utilization in high-risk patients. The integration of inflammation-based scores with CAR-IMPI showed orthogonal prognostic utility. Future research should prospectively assess the value of CAR-IMPI in clinical practice.

## Supplementary information

Supplemental Table S1

Supplemental Table S2

Supplemental Table S3

Supplemental Table S4

Supplemental Table S5

Supplemental Methods and Figures

## Data Availability

The datasets generated during and/or analyzed during the current study are available from the corresponding author on reasonable request.
