# Supplementary material for: Optimization and validation of the international metabolic prognostic index for CD19 CAR-T in large B-cell lymphoma
Source: Blood Cancer J. 2025 Aug 26;15(1):144. doi: 10.1038/s41408-025-01338-1 (PMC12381142; doi:10.1038/s41408-025-01338-1)
Supplement: Supplementary file 1 — Supplemental Table S1 [file 41408_2025_1338_MOESM1_ESM.docx]

**Table S1: Cohort Level Description of Key Clinical Outcomes.**

| **Characteristic** | **All patients**  **(n = 504)^1^** | **Development**  **(n = 256)^1^** | **Validation**  **(n = 248)^1^** |
| --- | --- | --- | --- |
| **Toxicity** | | | |
| CRS Grade  0  1  2  3  4  5  Unknown | 83 (17%)  208 (43%)  164 (34%)  22 (4.5%)  7 (1.4%)  3 (0.6%)  17 | 37 (14%)  123 (48%)  79 (31%)  11 (4.3%)  4 (1.6%)  2 (0.8%)  0 | 46 (20%)  85 (37%)  85 (37%)  11 (4.8%)  3 (1.3%)  1 (0.4%)  17 |
| ICANS Grade  0  1  2  3  4  5  Unknown | 281 (60%)  60 (13%)  52 (11%)  53 (11%)  19 (4.1%)  2 (0.4%)  38 | 142 (56%)  37 (15%)  33 (13%)  28 (11%)  14 (5.5%)  0 (0%)  2 | 139 (66%)  23 (11%)  18 (8.5%)  25 (12%)  5 (2.4%)  2 (0.9%)  36 |
| ICU Admission  Unknown | 68 (14%)  2 | 33 (13%)  1 | 36 (15%)  1 |
| Treatment with Tocilizumab | 234 (46%) | 128 (50%) | 106 (43%) |
| Treatment with Steroids  Unknown | 191 (38%)  4 | 105 (42%)  4 | 86 (35%)  0 |
| **Survival and follow-up** | | | |
| Median follow-up (mo) | 32.1 (30.0, 35.5) | 27.6 (24.2, 30.0) | 45.4 (37.5, 51.5) |
| Median PFS (mo) | 7.6 (5.7, 10.6) | 7.9 (5.0, 14.3) | 7.5 (5.3, 11.9) |
| Median OS (mo) | 34.0 (25.9, 63.4) | 33.4 (22.6, not- reached) | 34.0 (22.8, not- reached) |
