# Supplementary material for: Optimization and validation of the international metabolic prognostic index for CD19 CAR-T in large B-cell lymphoma
Source: Blood Cancer J. 2025 Aug 26;15(1):144. doi: 10.1038/s41408-025-01338-1 (PMC12381142; doi:10.1038/s41408-025-01338-1)
Supplement: Supplementary file 2 — Supplemental Table S2 [file 41408_2025_1338_MOESM2_ESM.docx]

**Table S2: Patient characteristics by median CAR-IMPI**

|  | **All patients**  **(n = 504)^1^** | **Low**  **(n = 293)^1^** | **High**  **(n = 211)^1^** |
| --- | --- | --- | --- |
| **Patient Demographics** | | | |
| Median age (range) | 65 (56, 71) | 63 (52, 71) | 67 (60, 71) |
| Sex  Female  Male | 188 (37%)  316 (63%) | 118 (40%)  175 (60%) | 70 (33%)  141 (67%) |
| ECOG Score  >1  0-1 | 70 (14%)  434 (86%) | 16 (5%)  277 (95%) | 54 (26%)  157 (74%) |
| **Treatment-related Features** | | | |
| CAR-T Product  Axicabtagene ciloleucel  Lisocabtagene maraleucel  Tisagenlecleucel | 285 (57%)  70 (14%)  149 (30%) | 168 (57%)  43 (15%)  82 (28%) | 117 (55%)  27 (13%)  67 (32%) |
| Number of Prior Treatment Lines (excluding Bridging) | 2 (1, 3) | 2 (1, 3) | 2 (2, 3) |
| Underwent Bridging Therapy | 386/502 (77%) | 208/254 (71%) | 178/211 (84%) |
| **Disease Features** | | | |
| Primary Refractory Disease | 202/503 (40%) | 111/292 (38%) | 91/211 (43%) |
| Post-Bridging Bulky Disease (>10 cm) | 46/436 (11%) | 13/242 (5.4%) | 33/194 (17%) |
| Baseline MTV | 49 ml (5, 223) | 10 ml (1, 32) | 264 ml (129, 569) |
| Prior Autologous HCT | 111 (22%) | 76 (26%) | 35 (17%) |
| Ann Arbor Stage  0-2  3-4 | 133 (26%)  371 (74%) | 119 (41%)  174 (59%) | 14 (6.6%)  197 (93%) |
| Transformed Disease | 159/503 (32%) | 78/293 (27%) | 81/210 (39%) |
| Double/Triple Hit | 109/364 (30%) | 57/213 (27%) | 52/151 (34%) |
| **Laboratory Findings (Pre-LD)** | | | |
| Baseline LDH (U/L) | 253 (193, 400) | 222 (179, 286) | 377 (247, 638) |
| Baseline Hemoglobin (g/dL) | 10.50 (9.30, 11.80) | 10.6 (9.30, 11.80) | 9.90 (8.70, 10.90) |
| Baseline Platelets (10^9^/L) | 168 (113, 219) | 171 (137, 214) | 161 (95, 227) |
| Baseline ANC (10^9^/L) | 3.10 (1.91, 4.60) | 3.00 (1.90, 4.20) | 3.30 (1.95, 5.06) |
| Baseline Ferritin (ng/mL)* | 349 (107, 812) | 243 (75, 512) | 535 (226, 1,241) |
| Baseline CRP (mg/dL)* | 1.0 (0.4, 3.4) | 0.5 (0.2, 1.5) | 2.7 (1.0, 7.6) |
| Hematotox Score  High  Low  Unknown* | 209 (42%)  285 (58%)  10 | 87 (31%)  196 (69%)  10 | 122 (58%)  89 (42%)  0 |
| ^1^ Median (Q1, Q3); n (%). The denominator has been included in case of missing values.  ^*^ CRP and Ferritin values missing in 5 patients in the development cohort, respectively. CAR-HEMATOTOX scores were evaluable in 494 patients and calculated according to *Rejeski et al, Blood 2021*).  Abbreviations: ECOG, Eastern Cooperative Oncology Group. MTV, metabolic tumor volume. HCT, hematopoietic cell transplantation. LD, lymphodepletion (typically day -5 before CAR T-cell infusion). LDH, Lactate Dehydrogenase. ANC, absolute neutrophil count. CRP, C-reactive protein. | | | |
