# Supplementary material for: Optimization and validation of the international metabolic prognostic index for CD19 CAR-T in large B-cell lymphoma
Source: Blood Cancer J. 2025 Aug 26;15(1):144. doi: 10.1038/s41408-025-01338-1 (PMC12381142; doi:10.1038/s41408-025-01338-1)
Supplement: Supplementary file 3 — Supplemental Table S3 [file 41408_2025_1338_MOESM3_ESM.docx]

**Table S3: Patient characteristics by CAR-IMPI terciles**

|  | **Low**  **(n = 206)^1^** | **Intermediate**  **(n = 157)^1^** | **High**  **(n = 141)^1^** |
| --- | --- | --- | --- |
| **Patient Demographics** | | | |
| Median age (range) | 63 (52, 71) | 63 (56, 69) | 68 (61, 74) |
| Sex  Female  Male | 85 (41%)  121 (59%) | 61 (39%)  96 (61%) | 42 (30%)  99 (70%) |
| ECOG Score  >1  0-1 | 12 (5.8%)  194 (94%) | 18 (11%)  139 (89%) | 40 (28%)  101 (72%) |
| **Treatment-related Features** | | | |
| CAR-T Product  Axicabtagene ciloleucel  Lisocabtagene maraleucel  Tisagenlecleucel | 119 (58%)  28 (14%)  59 (29%) | 93 (59%)  20 (13%)  44 (28%) | 73 (52%)  22 (16%)  46 (33%) |
| Number of Prior Treatment Lines (excluding Bridging) | 2 (1, 3) | 2 (1, 3) | 2 (2, 4) |
| Underwent Bridging Therapy | 147/204 (72%) | 117/157 (75%) | 122/141 (87%) |
| **Disease Features** | | | |
| Primary Refractory Disease | 70/205 (34%) | 70/157 (45%) | 62/141 (44%) |
| Post-Bridging Bulky Disease (>10 cm) | 6/173 (3.5%) | 10/132 (7.6%) | 30/131 (23%) |
| Baseline MTV | 3 (0, 11) | 74 (42, 131) | 455 (241, 735) |
| Prior Autologous HCT | 51 (25%) | 40 (25%) | 20 (14%) |
| Ann Arbor Stage  0-2  3-4 | 82 (40%)  124 (60%) | 44 (28%)  113 (72%) | 7 (5.0%)  134 (95%) |
| Transformed Disease | 53/206 (26%) | 52/156 (33%) | 54/141 (38%) |
| Double/Triple Hit | 34/152 (22%) | 42/108 (39%) | 33/104 (32%) |
| **Laboratory Findings (Pre-LD)** | | | |
| Baseline LDH (U/L) | 203 (168, 259) | 260 (211, 356) | 476 (294, 738) |
| Baseline Hemoglobin (g/dL) | 11.50 (10.10, 12.60) | 10.40 (9.10, 11.80) | 9.60 (8.50, 10.70) |
| Baseline Platelets (10^9^/L) | 173 (137, 214) | 169 (113, 217) | 160 (89, 224) |
| Baseline ANC (10^9^/L) | 3.00 (1.90, 4.30) | 3.15 (1.99, 4.60) | 3.20 (1.92, 5.09) |
| Baseline Ferritin (ng/mL)* | 203 (64, 425) | 325 (145, 725) | 678 (354, 1,491) |
| Baseline CRP (mg/dL)* | 0.5 (0.2, 1.2) | 1.2 (0.5, 3.0) | 3.3 (1.3, 8.1) |
| Hematotox Score  High  Low  Unknown* | 52 (26%)  145 (74%)  9 | 66 (42%)  90 (58%)  1 | 91 (65%)  50 (35%)  0 |
| ^1^ Median (Q1, Q3); n (%). The denominator has been included in case of missing values.  ^*^ CRP and Ferritin values missing in 5 patients in the development cohort, respectively. CAR-HEMATOTOX scores were evaluable in 494 patients and calculated according to *Rejeski et al, Blood 2021*).  Abbreviations: ECOG, Eastern Cooperative Oncology Group. MTV, metabolic tumor volume. HCT, hematopoietic cell transplantation. LD, lymphodepletion (typically day -5 before CAR T-cell infusion). LDH, Lactate Dehydrogenase. ANC, absolute neutrophil count. CRP, C-reactive protein. | | | |
