# Supplementary material for: Optimization and validation of the international metabolic prognostic index for CD19 CAR-T in large B-cell lymphoma
Source: Blood Cancer J. 2025 Aug 26;15(1):144. doi: 10.1038/s41408-025-01338-1 (PMC12381142; doi:10.1038/s41408-025-01338-1)
Supplement: Supplementary file 4 — Supplemental Table S4 [file 41408_2025_1338_MOESM4_ESM.docx]

**Table S4: Multivariate Cox Regression for Overall Survival**

|  | Development | | | Validation | | |
| --- | --- | --- | --- | --- | --- | --- |
| Characteristic | **N** | **HR (95%-CI)** | **p** | **N** | **HR (95%-CI)** | **p** |
| CAR-IMPI  (continuous) | 256 (100%) | 1.98  (1.21 – 3.24) | **0.006** | 248 (100%) | 1.69  (1.41 - 2.02) | **1.3 x 10^-8^** |
| CAR-T Product |  |  |  |  |  |  |
| Axi-cel | 155 (61%) | Ref. |  | 130 (52%) | Ref. |  |
| Liso-cel | 19 (7.4%) | 1.48  (0.68 – 3.21) | 0.326 | 51 (21%) | 1.13  (0.69 - 1.83) | 0.630 |
| Tisa-cel | 82 (32%) | 1.14  (0.70 – 1.88) | 0.596 | 67 (27%) | 2.04  (1.43 - 2.91) | **2.7 x 10^-4^** |
| ECOG |  |  |  |  |  |  |
| 0-1 | 220 (86%) | Ref. |  | 163 (66%) | Ref. |  |
| 2-4 | 36 (14%) | 1.64  (1.20 – 2.23) | **0.002** | 85 (34%) | 1.31  (0.93 - 1.85) | 0.120 |
| LDH  (>ULN) | 256 (100%) | 1.70  (1.01 - 2.86) | **0.047** | 248 (100%) | 2.19  (1.47 - 3.27) | **1.1 x 10^-4^** |
| CRP  (continuous) | 251 (98%) | 1.02  (1.00 - 1.04) | **0.026** | 248 (100%) | 1.04  (1.00 - 1.08) | **0.027** |
| Center* | * | * | * | - | - | - |
| The output of the multivariable Cox Regression model for progression-free survival is provided for the development and validation cohort – performed separately. P-values reaching statistical significance (p<0.05) are highlighted in bold. The number of patients (N) in each strata and respective reference (Ref.) variable are depicted. All laboratory values were determined before lymphodepletion with a leniency period of 5 days.  ^*^ The center variable was introduced into the multivariable model as a stratification variable (not applicable to the monocentric validation cohort).  Abbreviations: HR, hazard ratio. CI, confidence interval. CAR, chimeric antigen receptor. IMPI, international metabolic prognostic index. Axi-cel, axicabtagene ciloleucel. Liso-cel, lisocabtagen maraleucel. Tisa-cel, tisagenlecleucel. ECOG, Eastern Cooperative Oncology Group. LDH, Lactate Dehydrogenase. ULN, upper limit of normal. CRP, C-reactive protein. | | | | | | |
