# Supplementary material for: Optimization and validation of the international metabolic prognostic index for CD19 CAR-T in large B-cell lymphoma
Source: Blood Cancer J. 2025 Aug 26;15(1):144. doi: 10.1038/s41408-025-01338-1 (PMC12381142; doi:10.1038/s41408-025-01338-1)
Supplement: Supplementary file 5 — Supplemental Table S5 [file 41408_2025_1338_MOESM5_ESM.docx]

**Table S5: Key Clinical Outcomes stratified by CAR-IMPI.**

| **Characteristic** | **Low**  **(n = 205)^1^** | **Intermediate**  **(n = 157)^1^** | **High**  **(n = 142)^1^** |
| --- | --- | --- | --- |
| **Toxicity** | | | |
| CRS Grade  0  1  2  3  4  5  Unknown | 21 (21%)  81 (41%)  72 (36%)  2 (1.0%)  2 (1.0%)  0 (0%)  6 | 25 (17%)  75 (50%)  42 (28%)  7 (4.7%)  0 (0%)  0 (0%)  8 | 16 (12%)  52 (37%)  50 (36%)  13 (9.4%)  5 (3.6%)  3 (2.2%)  3 |
| ICANS Grade  0  1  2  3  4  5  Unknown | 130 (69%)  19 (10%)  16 (8.5%)  14 (7.4%)  9 (4.8%)  0 (0%)  17 | 92 (63%)  18 (12%)  12 (8.2%)  19 (13%)  5 (3.4%)  0 (0%)  11 | 59 (45%)  23 (17%)  23 (17%)  20 (15%)  5 (3.8%)  2 (1.5%)  10 |
| ICU Admission  Unknown | 18 (8.8%)  1 | 16 (10%)  1 | 35 (25%)  0 |
| Treatment with Tocilizumab | 92 (45%) | 64 (41%) | 78 (55%) |
| Treatment with Steroids  Unknown | 70 (34%)  1 | 53 (34%)  2 | 68 (48%)  1 |
