# Supplementary material for: Optimization and validation of the international metabolic prognostic index for CD19 CAR-T in large B-cell lymphoma
Source: Blood Cancer J. 2025 Aug 26;15(1):144. doi: 10.1038/s41408-025-01338-1 (PMC12381142; doi:10.1038/s41408-025-01338-1)
Supplement: Supplementary file 6 — Supplemental Methods and Figures [file 41408_2025_1338_MOESM6_ESM.pdf]

SUPPLEMENTARY METHODS

The formula for calculation of CAR-IMPI can be summarized as follows:

'CAR-IMPI' = 0.0149 x MTV_a (mL) + 0.0000870 x MTV_b (mL) + 0.00817 x age (years) + Ann Arbor Stage Coefficient;

where if MTV $\leq$ 44.3, MTV_a = MTV and MTV_b = 0 and if MTV > 44.3, MTV_a=44.3 and MTV_b=MTV-44.3.

Ann Arbor Stage Coefficient = 0 for Stage 1, - 0.0402 for Stage 2, 0.271 for Stage 3, or 0.219 for Stage 4.

SUPPLEMENTARY FIGURES

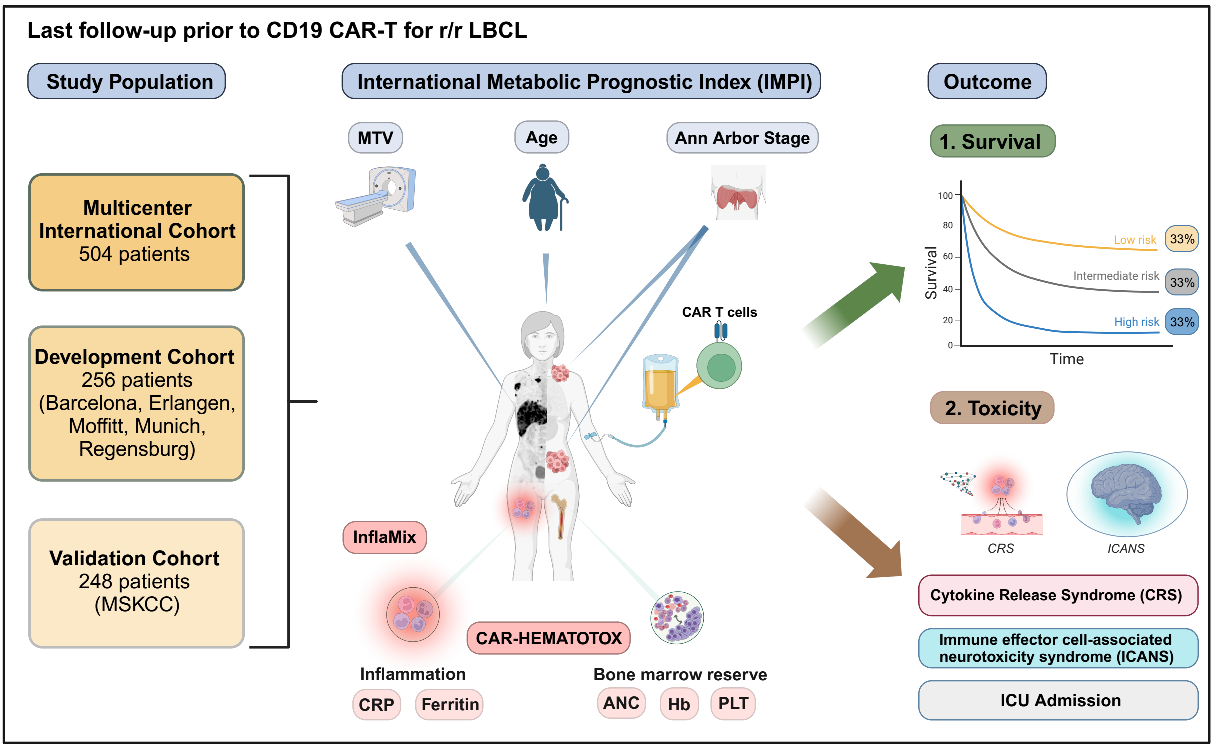

Figure S1. Study Design. Presented is an overview of the composition of the 504 patients included in this study. Of these, 256 patients were in the development cohort and 248 patients in the validation cohort. The middle part of the figure displays the components used to calculate the International Metabolic Prognostic Index (IMPI) and CAR-HEMATOTOX at the last time point prior to CAR-T cell therapy. Outcomes parameters analyzed were progression-free survival (PFS) and overall survival (OS) based on the three equal-sized CAR-IMPI-based risk groups. In the second part, evaluations were carried out to analyze frequency and severity of cytokine release syndrome (CRS) and Immune effector cell-associated neurotoxicity syndrome (ICANS), and frequency of ICU admission.

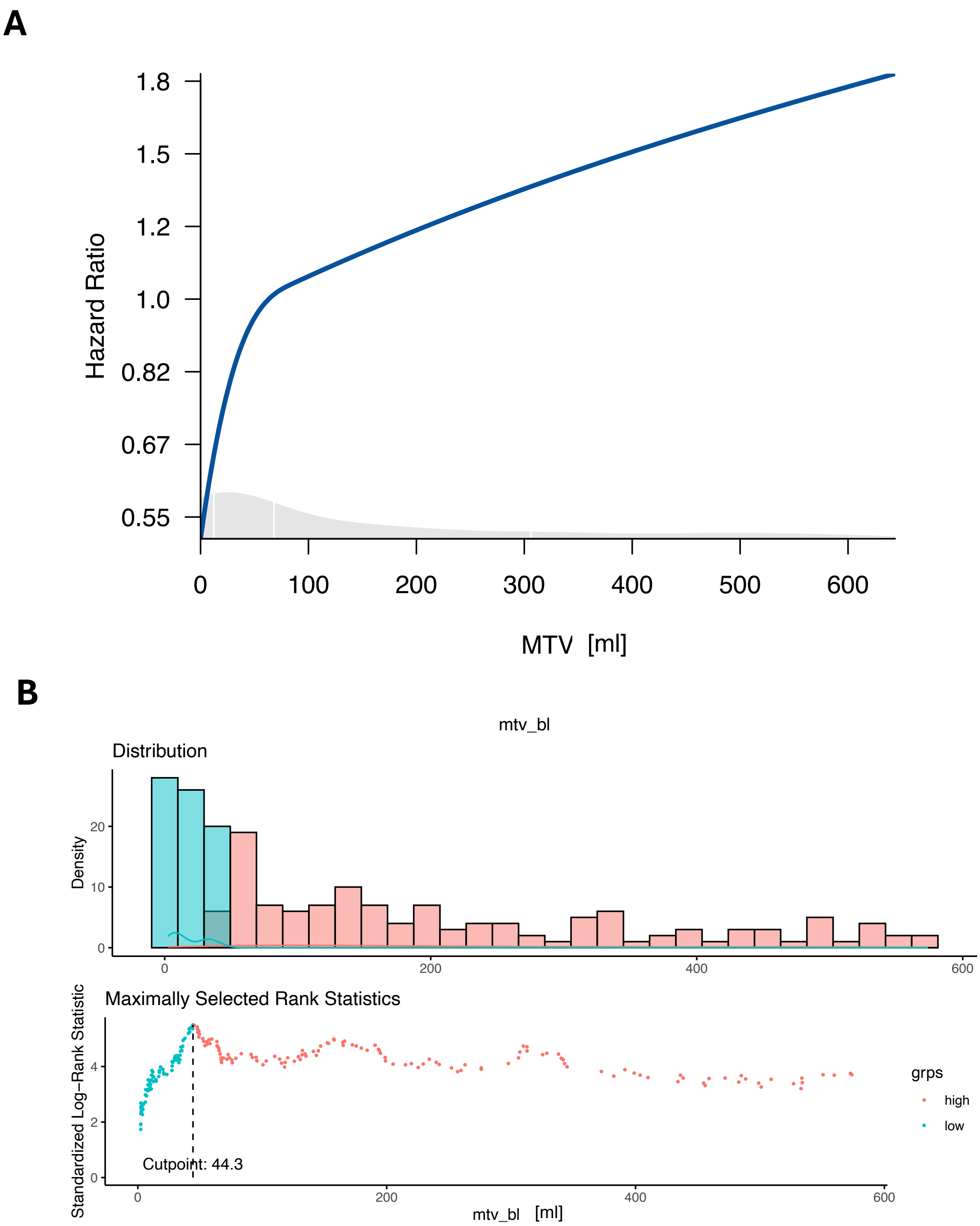

Figure S2. Restricted Cubic Spline and Max Stat.
Panel A: Restricted cubic spline with 4 knots examining the association between metabolic tumor volume (MTV) and the hazard ratio for progression-free survival (PFS). **Panel B:** Maximally selected rank statistics analyzing the relationship between baseline MTV and PFS. The identified optimal MTV threshold of 44.3 mL is similar to the graphical inflection point at approximately 50 mL observed in the restricted cubic spline analysis (panel A).

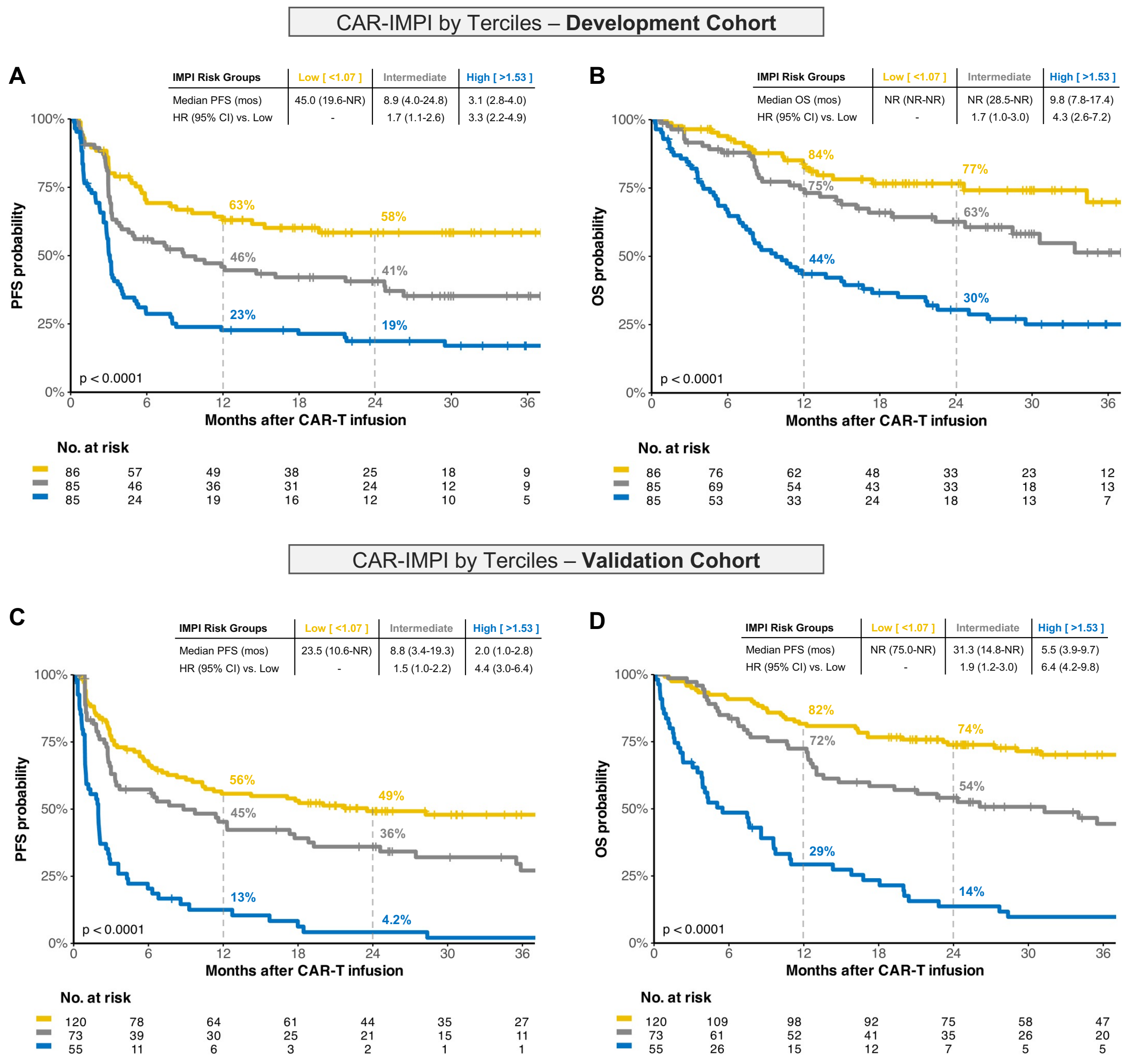

Figure S3. Survival analysis stratified by CAR-IMPI risk groups. The top row shows the survival curves for PFS (**A**) and OS (**B**) in the development cohort according to the CAR-IMPI-based risk groups. The bottom section shows the survival curves for PFS (**C**) and OS (**D**) using the same stratification with the same CAR-IMPI cut-offs in the validation cohort. The low-risk group is marked in yellow, the intermediate-risk group in gray and the high-risk group in blue. The hazard ratio (HR) with 95% confidence interval (CI) from the univariate Cox regression using the low-risk group as reference is provided.

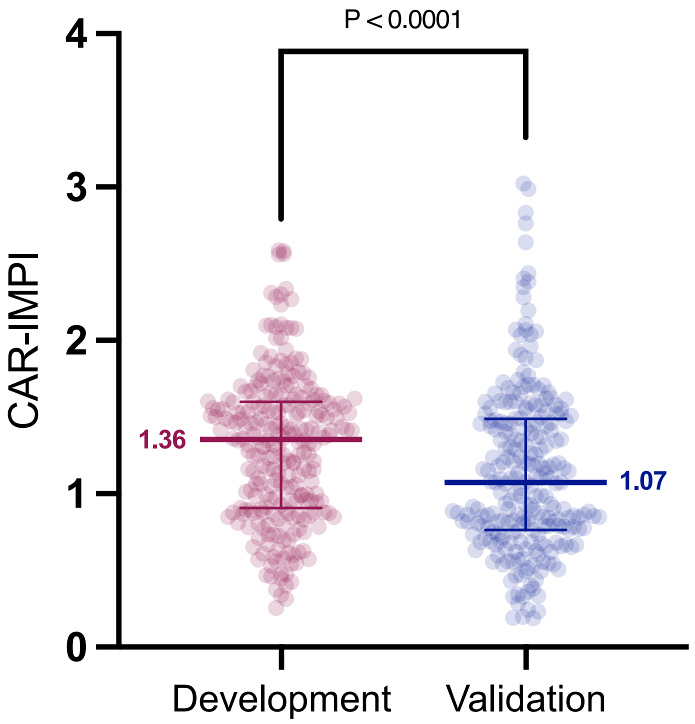

Figure S4. CAR-IMPI by patient cohort.
Scatter dot plot comparing absolute CAR-IMPI values per patient in the development (magenta) versus validation (blue) cohort. Line and whiskers indicate the median with interquartile range. The p-value of the Mann-Whitney test is provided.
